# Optimization of Antibacterial Activity of *Perilla frutescens var. acuta* Leaf against *Pseudomonas aeruginosa* Using the Evolutionary Operation-Factorial Design Technique

**DOI:** 10.3390/ijms11103922

**Published:** 2010-10-14

**Authors:** Ung-Kyu Choi, Ok-Hwan Lee, Seong-Il Lim, Young-Chan Kim

**Affiliations:** 1 Pohang Center for Evaluation of Biomaterials, Pohang 790-834, Korea; E-Mail: cuk8272@hanmail.net; 2 Department of Food Science and Biotechnology, Kangwon national University, Chuncheon, 200-701, Korea; 3 Korea Food Research Institute, Seongnam, Kyonggi, 463-746, Korea; E-Mail: silim@kfri.re.kr

**Keywords:** *Perilla frutescens var. acuta leaf*, EVOP-factorial design technique, antibacterial activity, *Pseudomonas aeruginosa* KCTC 2004

## Abstract

This study was undertaken to optimize the conditions for the extraction of antibacterial activity of *Perilla frutescens var. acuta* leaf against *Pseudomonas aeruginosa* KCTC 2004 using the evolutionary operation-factorial (EVOP) design technique. Increased antibacterial activity was achieved at higher extraction temperatures and with a longer extraction time. Antibacterial activity was not affected by differing ethanol concentration in the extraction solvent. The maximum antibacterial activity of ethanolic extract of *P. frutescens var. acuta* leaf against *P. aeruginosa*, determined by the EVOP factorial technique, was obtained at an extraction temperature of 80 °C (R = −0.800^**^), 26 h (R = −0.731^**^) extraction time, and 50% (R = −0.075) ethanol concentration. The population of *P. aeruginosa* also decreased from 6.660 log CFU/mL in the initial set to 4.060 log CFU/mL in the third set. Also, scanning electron microscopy study of the ethanolic extract of *P. frutescens var. acuta* revealed potential detrimental effects on the morphology of *P. aeruginosa*.

## 1. Introduction

With increasing bacterial resistance to antibiotics, there is considerable interest in investigating the antimicrobial effects of plant extracts against a range of bacteria, to develop other classes of safe and natural antimicrobials useful for infection control or for the preservation of food [1]. *Pseudomonas aeruginosa* (KCTC 2004) is a versatile pathogen associated with a broad spectrum of infections in human beings and the food industry. *P. aeruginosa* dominates proteinaceous foods stored aerobically at chilled temperatures. In pasteurized milk it originates from post-process contamination [2] and spoils the food products. *Pseudomonas* sp. is widespread in the environment [3,4] and is often responsible, as an opportunistic bacterium, for very serious episodes of infections. *P. aeruginosa* is also involved in infections ranging widely in location and severity from soft tissue abscesses, bronchopneumonia, otitis externa and otitis media to chronic pneumopathy or mucoviscidosis [5]. This organism has been shown to possess a remarkable capacity to resist antibiotics, either intrinsically or following acquisition of resistance genes [6].

The main advantage of the evolutionary operation (EVOP) factorial design technique [7–9] is to develop more effective approaches for the optimization of an ‘n’ variable system [10,11], using EVOP methodology including response surface methodology (RSM) derived from orthogonal polynomial fitting techniques [12,13]. The identification and evaluation of natural products with optimized levels of antimicrobial activity for the control of infectious pathogens can be considered as an important international challenge for the food and medicine industries.

The *Perilla frutescens* L. herb belongs to the family Labiatae. *P. frutescens var. acuta* is an edible plant frequently used as one of the most popular garnishes and food colorants in China, Japan and Korea. The leaves of *P. frutescens var. acuta* have been shown to be detoxicant, antitussive, antibiotic, and antipyretic [14,15], and are also utilized as a folk medicine for treating intestinal disorders and allergies, particularly in traditional Chinese medical practice [16]. Although the biological activity of *P. frutescens var. acuta* is well documented, there is no report available on the optimization of the antibacterial activity of the *P. frutescens var. acuta* leaf against *P. aeruginosa* using the EVOP-factorial design technique.

In the present study, therefore, the optimal conditions of extraction temperature, extraction time and ethanol concentration on the antibacterial activity of *Perilla frutescens var. acuta* leaf were investigated using the EVOP-factorial design to maximize the antibacterial activity of *Perilla frutescens var. acuta* leaf against *P. aeruginosa*.

## 2. Results and Discussion

### 2.1. Optimization of Antibacterial Activity by EVOP-Factorial System

The experimental conditions used in the first set of experiments, the corresponding antibacterial activities of cycle I and II, their differences and average values are presented in Table 1. The error limits, effects and the change in the mean effect were calculated and the results are shown in Table 2. The extraction temperature, extraction time and ethanol concentration of the central point in first set (E_10_, E_20_) were 50 °C, 14 h and 50%, respectively.

In the first set, the error limits for average, effects and changes in mean were 0.1739, 0.1235 and 0.1096, respectively. The change in mean effect was −0.2105. According to the decision-making procedure, after calculating the change in the mean effect and error limit, an examination was necessary to determine whether any change in the control (search level) experimental conditions would help to improve the objective function [10]. The optimum condition was achieved when the effect was smaller than the error limit while the change in the mean effect was large. Moreover, because the dependent variables are the population of *P. aeruginosa* in which growth was suppressed by addition of *P. frutescens var. acuta* leaf extract, the optimum point was reached when the code of mean effect was negative.

The determination of the magnitude of the change in mean effect, which is negative and large, compared to the error limit, is a requirement in order to confirm the achievement of the optimum condition. Such a situation, where some of the effects are larger in comparison to the error limit, does not ensure that the conditions in the search region (E_10_, E_20_) of the first set is the actual optimum and a second set of experiments is called for.

In the second set, the search level (E_10_, E_20_) was fixed at the best condition of Set I, at a level of E_21_, in which the number of *P. aeruginosa* was 4.955 log CFU/mL. The extraction temperature, extraction time and ethanol concentration of the central point in the second set (E_10_, E_20_) were 65 °C, 20 h and 75%, respectively. The experimental conditions and the results of the Set II experiments are presented in Table 3 and the effects and error limits are shown in Table 4. In the second set, the error limits for average, effects and changes in mean was 0.1612, 0.1145 and 0.1016, respectively. The change in mean effect was −0.0685. The most effective antibacterial activity (4.095 log CFU/mL) was obtained at E_14_. The extraction temperature, extraction time and ethanol concentration of the E_14_ point in the second set were 80 °C, 26 h and 50%, respectively. In this case, not all of the effects were smaller than the error limit, and the change in mean effect was smaller compared to the error limit even though it is positive. It has been reported that if all or any of the effects are larger than the error limits, the change in the experimental conditions may yield better results [10].

**Figure 1:**
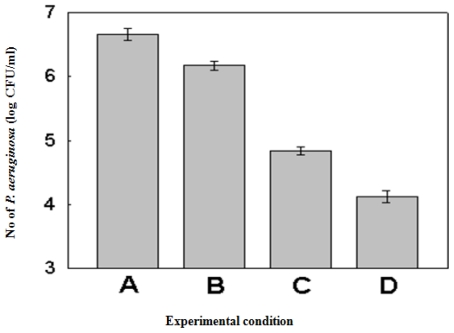
Comparison of the antibacterial activity of *Perilla frutescens var. acuta* leaf against *P. aeruginosa* at the central point of each set. (**A**) E_11_ of Set I (extraction temperature; 35 °C, extraction time; 8 h, ethanol concentration; 25%); (**B**) central point of Set I (Extraction temperature; 50 °C, extraction time; 14 h, ethanol concentration; 50%); (**C**) central point of Set II (Extraction temperature; 65 °C, extraction time; 20 h, ethanol concentration; 75%); (**D**) central point of Set III (Extraction temperature; 80 °C, extraction time; 26 h, ethanol concentration; 50%).

From the above situation, the third set of experiments was designed in which the best condition of Set II (E_14_) was selected as the search level (E_10_, E_20_) for Set III. The experimental conditions and the results of Set III are shown in Table 5 and the calculated effects and error limits are presented in Table 6. In the EVOP-factorial design, the effects remain smaller than the error limits while the changes in the mean effect remain larger and positive so as to reach the optimum level. Thus, in the experiments of third set, we were able to arrive at the proper optimum condition, in which all effects were smaller than the error limit and the change in mean effect is large and positive. As shown in Figure 1, the population of *P. aeruginosa* decreased from 6.660 log CFU/mL in the initial set to 4.060 log CFU/mL in the third set.

**Figure 2:**
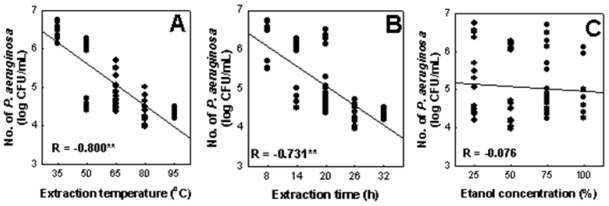
Plots of the responses against the three independent variables in EVOP. ** *p* < 0.01. (**A**) Extraction temperature; (**B**) extraction time; (**C**) ethanol concentration.

In this study, it was shown that higher antibacterial activity was achieved at a higher extraction temperature of 80 °C (R = −0.800^**^) and in a longer extraction time of 26 h (R = −0.731^**^). However, antibacterial activity of *P. frutescens var. acuta* leaf extract against *P. aeruginosa* was not affected by the presence of different ethanol concentrations in the extraction solvent (R = −0.076), as shown in Figure 2. Therefore, the maximum antibacterial activity of *P. frutescens var. acuta* leaf against *P. aeruginosa*, determined by the EVOP-factorial technique, was obtained at 80 °C extraction temperature, 26 h extraction time and 50% ethanol concentration. These conditions were also found in our previous study conducted on *Coptidis rhizoma* against *Streptococcus mutans* [17].

Although the optimal condition of *P. frutescens var. acuta* on antibacterial activity is well described in this study, the extraction yield and bioactive compounds in *P. frutescens var. acuta* are highly variable depending on environmental conditions. Therefore, further studies are needed to compare the effects of various seasonal samples of *P. frutescens var. acuta* leaf on antibacterial activity using EVOP-factorial design technique.

### 2.2. Scanning Electron Microscopy (SEM)

**Figure 3:**
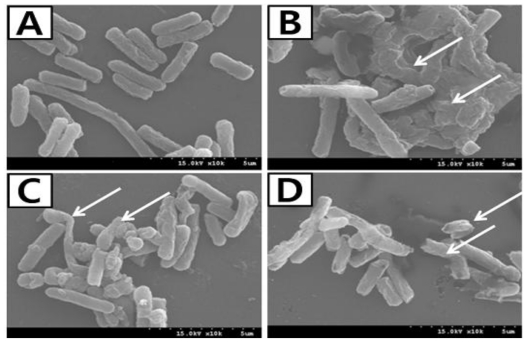
Scanning electron micrographs of *P. aeruginosa* incubated with the optimum concentration of ethanolic leaf extract of *Perilla frutescens var. acuta* (**A**) Control (absence of extract); (**B**) disruption and lysis of membrane integrity; (**C**) wrinkled abnormalities and cleft formation; (**D**) abnormal breaking of cell.

Further, a SEM study of was carried out to visualize the effects of the ethanolic extract of *P. frutescens var. acuta* leaf on the morphology of *P. aeruginosa* and demonstrated altered cell morphology as compared to a control group (Figure 3). Control cells in the absence of the extract showed a regular, smooth surface (Figure 3A). In contrast, cells inoculated with the ethanolic extract of *P. frutescens var. acuta* leaf revealed severe detrimental effects on the morphology of the cell membrane, showing disruption and lysis of the membrane integrity (Figure 3B). Exposure of *P. frutescens* leaf extract to *P. aeruginosa* revealed extensive surface collapse and wrinkled abnormalities on the morphology of the cells along with some small clefts formation (Figure 3C, 3D) and these finding are in strong agreement with a previous report [16]. Besides, several researchers have reported the effects of various plant extracts on the morphology of pathogenic bacteria including *P. aeruginosa* [18,19].

## 3. Experimental Section

### 3.1. Plant Material

The leaves of *P. frutescens var. acuta* were obtained from Yakrung market, Daegu, Republic of Korea, in July 2008. The specimen was lyophilized for 48–72 h after storage at −70 °C. Freeze dried samples were pulverized with a blender (HJM-7000, Hanil, Korea). Extra pure grade ethanol solvent was purchased from Daemyung Scientific Co., Daegu, Republic of Korea. Chemical reagents were obtained from Sigma Co. (St. Louis, MO, USA), unless otherwise stated.

### 3.2. Microorganism

*Pseudomonas aeruginosa* KCTC 2004 was used in the antibacterial assay. The strain was obtained from the Korea Food and Drug Administration (KFDA), Daegu, Republic of Korea. Active cultures for experimental use were prepared by transferring a loopful of cells from stock cultures to flasks and inoculatino in Luria-Bertani (LB) broth medium at 37 °C for 24 h. A culture of the bacterial strain was maintained on LB agar medium at 4 °C.

### 3.3. Preparation of Extracts from Various Conditions

To design an experiment for establishing proper extraction conditions, 20 g of sample was hydrolyzed and extracted in a reflux extraction apparatus under different extraction temperatures (35, 50, 65, 80 and 95 °C), extraction times (8, 14, 20, 26 and 32 h) and ethanol concentrations (25, 50, 75 and 100%) under EVOP factorial design and then freeze dried in order to establish the proper extraction conditions. The concentration of *P. frutescens var. acuta* extract used for the determination of the microbial population was 0.1%. Samples were homogenized for 30 seconds and serially diluted with peptone water (Difco, USA), as needed for the determination of microbial populations.

### 3.4. EVOP-Factorial Design Technique

In this study, the optimization of antibacterial activity of *Perilla frutescens var. acuta* leaf was investigated using the EVOP-factorial design technique, which was first proposed for the optimization of different operational parameters in a functioning chemical processing plant. The main advantage of EVOP methodology is its clear-cut decision making procedure, which indicates the change in influencing variables toward the desired maxima or minima [20]. The EVOP-factorial design technique was applied to select the optimum conditions of three extraction factors (temperature, time, and ethanol concentration) in different experiments [9]. Firstly, the control or search level experimental conditions (E_10_, E_20_) were selected based on the results of earlier investigation on the effect of individual extraction conditions on the antibacterial activity of the ethanolic extract of *P. frutescens var. acuta* leaf [21]. Secondly, the new experimental conditions (E_be_) were selected with lower and higher levels of inducers compared to the initial search level (E_b0_). Antibacterial activities of *P. frutescens var. acuta* leaf extract were estimated following the given assay procedure and recorded for cycle I and II. Differences in the antibacterial activities between cycle I and II, and average antibacterial activities were calculated to estimate the effects and error limits. The magnitudes of effects, error limits and change in mean effect were examined as per the decision making procedure to arrive at the optimum level. When the experimental results of the first set did not reach to the satisfactory level of optimum conditions, a second set of experiments was planned, selecting the best condition of the first set as the new search level for the second set. This procedure was repeated until the optimum condition was obtained.

Further, to determine the antibacterial activity of *P. frutescens var. acuta* leaf extract, enumeration of viable counts on LB plates was monitored as follows: 1 mL of the re-suspended culture was diluted into 9 mL buffer peptone water, thereby diluting it by 10-fold. In total, 0.1 mL sample of each treatment was diluted and spread on the surface of LB agar. The colonies were counted after 24 h of incubation at 37 °C.

### 3.5. Scanning Electron Microscopic (SEM) Analysis

To determine the efficacy of the extract of *P. frutescens var. acuta* leaf on the morphology of *P. aeruginosa*, SEM studies were performed using the optimum extraction conditions of the ethanolic extract of *P. frutescens var. acuta* leaf. Controls were prepared without extract. Further, to observe the morphological changes, the method of SEM was modified from Kockro *et al*.’s method [22]. The bacterial sample was washed gently with 50 mM/L phosphate buffer solution (pH 7.2), fixed with 2.5 g 100/mL glutaraldehyde and 1 g/100 mL osmic acid solution. The specimen was dehydrated using sequential exposure to ethanol concentrations ranging from 30–100%. The ethanol was replaced by tertiary butyl alcohol. After dehydration, the specimen was dried with CO_2_. Finally, the specimen was sputter-coated with gold in an ion coater for 2 min, followed by microscopic examinations (S-4300; Hitachi, Japan).

## 4. Conclusions

Overall, our results demonstrate that the application of EVOP-factorial design technique could serve as a potential tool to determine the optimum extraction conditions required to achieve the desired levels of antibacterial activity of natural products and their extracts for their potential utilization in the food industry to control food-borne pathogenic bacteria.

## Figures and Tables

**Table 1:** Experimental design for the three inducer system and results of Set I.

Experimental Conditions	E_10_^a^	E_11_	E_12_	E_13_	E_14_	E_20_	E_21_	E_22_	E_23_	E_24_
Temperature (°C)	50(0)^b^	35(−)	35(−)	65(+)	65(+)	50(0)	65(+)	35(−)	65(+)	35(−)
Time (h)	14(0)	8(−)	20(+)	8(−)	20(+)	14(0)	20(+)	8(−)	8(−)	20(+)
Ethanol concentration (%)	50(0)	25(−)	75(+)	75(+)	25(−)	50(0)	75(+)	75(+)	25(−)	25(−)
Antibacterial activity (cycle I) (log CFU/mL)	6.24	6.75	6.13	5.70	5.30	6.05	5.02	6.70	5.49	6.50
Antibacterial activity (cycle II) (log CFU/mL)	6.10	6.57	6.28	5.52	5.07	6.28	4.89	6.49	5.70	6.36
Difference (cycle I-cycle II) (log CFU/mL)	0.14	0.18	−0.15	0.18	0.23	0.23	0.13	0.21	−0.21	0.14
Average activity (log CFU/mL)	6.170 (a_10_)	6.660 (a_11_)	6.205 (a_12_)	5.610 (a_13_)	5.185 (a_14_)	6.165 (a_20_)	4.955 (a_21_)	6.595 (a_22_)	5.595 (a_23_)	6.430 (a_24_)

a E_10_ to E_24_ = experiments;

b Numbers in parentheses are the coded symbols of levels of the extraction conditions.

**Table 2:** Calculation worksheet of the effects of the three-variable system and magnitude of effects and error limits of Set I.

Experimental Conditions		Calculation of Effects
Temperature	1/4(a_13_ + a_14_ + a_21_ + a_23_ − a_11_ − a_12_ − a_22_ − a_24_)	−1.1363
Time	1/4(a_12_ + a_14_ + a_21_ + a_24_ − a_11_ − a_13_ − a_22_ − a_23_)	−0.4213
Ethanol concentration	1/4(a_12_ + a_13_ + a_21_ + a_22_− a_11_ − a_14_ − a_23_ − a_24_)	−0.1263
Temperature × time	1/4(a_11_ + a_14_ + a_21_ + a_22_ − a_12_ − a_13_ − a_23_ − a_24_)	−0.1113
Temperature × ethanol concentration	1/4(a_11_ + a_13_ + a_21_ + a_24_ − a_12_ − a_14_ − a_22_ − a_23_)	0.0188
Time × ethanol concentration	1/4(a_11_ + a_12_ + a_21_ + a_23_ − a_13_ − a_14_ − a_22_ − a_24_)	−0.1013
Temperature × time × ethanol concentration	1/4(a_21_ + a_22_ + a_23_ + a_24_ − a_11_ − a_12_ − a_13_ − a_14_)	−0.0213
Change in mean effect	1/10(a_11_ + a_12_ + a_13_ + a_14_ + a_21_ + a_22_ + a_23_ + a_24_ − 4a_10_ − 4a_20_)	−0.2105
Standard deviation (σ)	1/2(σ_1_ + σ_2_)=1/2(R_1_ × f_k,n_ + R_2_ × f_k,n_) ^(1)^	0.1230

Error limits:		
for average	±1.414σ (±2σ/√n)	0.1739
for effects	±1.004σ (±0.71 × 2σ/√n)	0.1235
for change in mean	±0.891σ (±0.63 × 2σ/√n)	0.1096

R_1_: (largest difference − smallest difference) in block 1; R_2_: (largest difference − smallest difference) in block 2; f_k,n_ = constant depending on number of replications (n) and number of experiments (k) per cycle = 0.3 for n = 2 and k = 5.

**Table 3:** Experimental design for three inducer system and results of Set II.

Experimental Conditions	E_10_	E_11_	E_12_	E_13_	E_14_	E_20_	E_21_	E_22_	E_23_	E_24_
Temperature (°C)	65(0)	50(−)	50(−)	80(+)	80(+)	65(0)	80(+)	50(−)	80(+)	50(−)
Time (h)	20(0)	14(−)	26(+)	14(−)	26(+)	20(0)	26(+)	14(−)	14(−)	26(+)
Ethanol concentration (%)	75(0)	50(−)	100(+)	100(+)	50(−)	75(0)	100(+)	100(+)	50(−)	50(−)
Antibacterial activity (cycle I) (log CFU/mL)	4.77	6.19	4.40	4.80	4.18	4.87	4.25	6.11	4.50	4.51
Antibacterial activity (cycle II) (log CFU/mL)	4.90	6.03	4.59	5.01	4.01	4.69	4.40	5.95	4.65	4.71
Difference (cycle I-cycle II) (log CFU/mL)	−0.13	0.16	−0.19	−0.21	0.17	0.18	−0.15	0.16	−0.15	−0.20
Average activity (log CFU/mL)	4.835 (a_10_)	6.110 (a_11_)	4.495 (a_12_)	4.905 (a_13_)	4.095 (a_14_)	4.780 (a_20_)	4.325 (a_21_)	6.030 (a_22_)	4.575 (a_23_)	4.610 (a_24_)

a E_10_ to E_24_ = experiments;

b Numbers in parentheses are the coded symbols of levels of the extraction conditions.

**Table 4:** Calculation worksheet of the effects of the three-variable system and magnitude of effects and error limits of Set II.

Experimental Conditions	Calculation of Effects
Temperature	−0.8363
Time	−1.0238
Ethanol concentration	0.0913
Temperature × time	0.4938
Temperature × ethanol concentration	0.1888
Time × ethanol concentration	−0.0338
Temperature × time × ethanol concentration	−0.0163
Change in mean effect	0.0685
Standard deviation (σ)	0.1140

Error limits:	
for average	0.1612
for effects	0.1145
for change in mean	0.1016

R_1_: (largest difference − smallest difference) in block 1; R_2_: (largest difference − smallest difference) in block 2; f_k,n_ = constant depending on number of replications (n) and number of experiments (k) per cycle = 0.3 for n = 2 and k = 5.

**Table 5:** Experimental design for the three inducer system and results of Set III.

Experimental Conditions	E_10_	E_11_	E_12_	E_13_	E_14_	E_20_	E_21_	E_22_	E_23_	E_24_
Temperature (°C)	80(0)	65(−)	65(−)	95(+)	95(+)	80(0)	95(+)	65(−)	95(+)	65(−)
Time (h)	26(0)	20(−)	32(+)	20(−)	32(+)	26(0)	32(+)	20(−)	20(−)	32(+)
Ethanol concentration (%)	50(0)	25(−)	75(+)	75(+)	25(−)	50(0)	75(+)	75(+)	25(−)	25(−)
Antibacterial activity (cycle I) (log CFU/mL)	4.21	4.38	4.51	4.41	4.4.	3.96	4.24	4.65	4.50	4.39
Antibacterial activity (cycle II) (log CFU/mL)	4.03	4.57	4.39	4.50	4.20	4.16	4.35	4.44	4.37	4.49
Difference (cycle I-cycle II) (log CFU/mL)	0.18	−0.15	0.12	−0.09	0.20	−0.20	−0.11	0.21	0.13	−0.10
Average activity (log CFU/mL)	4.120 (a_10_)	4.475 (a_11_)	4.450 (a_12_)	4.455 (a_13_)	4.300 (a_14_)	4.060 (a_20_)	4.295 (a_21_)	4.545 (a_22_)	4.435 (a_23_)	4.440 (a_24_)

a E_10_ to E_24_ = experiments;

b Numbers in parentheses are the coded symbols of levels of the extraction conditions.

**Table 6:** Calculation worksheet of the effects of the three-variable system and magnitude of effects and error limits of Set III.

Experimental Conditions	Calculation of Effects
Temperature	−0.1063
Time	−0.1063
Ethanol concentration	0.0238
Temperature × time	−0.0413
Temperature × ethanol concentration	−0.0163
Time × ethanol concentration	−0.0213
Temperature × time × ethanol concentration	0.0088
Change in mean effect	0.2675
Standard deviation (σ)	0.1140

Error limits:	
for average	0.1612
for effects	0.1145
for change in mean	0.1016

R_1_: (largest difference − smallest difference) in block 1; R_2_: (largest difference − smallest difference) in block 2; f_k,n_ = constant depending on number of replications (n) and number of experiments (k) per cycle = 0.3 for n = 2 and k = 5.
